# Strategies on Nanodiagnostics and Nanotherapies of the Three Common Cancers

**DOI:** 10.3390/nano8040202

**Published:** 2018-03-28

**Authors:** Fan Leng, Fang Liu, Yongtao Yang, Yu Wu, Weiqun Tian

**Affiliations:** Department of Biomedical Engineering, School of Basic Medical Sciences, Wuhan University, Wuhan 430071, China; fanleng@whu.edu.cn (F.L.); fangliu@whu.edu.cn (F.L.); yangyongtao@whu.edu.cn (Y.Y.); WuYu920202@hotmail.com (Y.W.)

**Keywords:** nanoparticles, nanodiagnostics, nanotherapies, common cancers

## Abstract

The emergence of nanomedicine has enriched the knowledge and strategies of treating diseases, and especially some incurable diseases, such as cancers, acquired immune deficiency syndrome (AIDS), and neurodegenerative diseases. The application of nanoparticles in medicine is in the core of nanomedicine. Nanoparticles can be used in drug delivery for improving the uptake of poorly soluble drugs, targeted delivery to a specific site, and drug bioavailability. Early diagnosis and targeted therapies for cancers can significantly improve patients’ quality of life and extend patients’ lives. The advantages of nanoparticles have given them a progressively important role in the nanodiagnosis and nanotherapy of common cancers. To provide a reference for the further application of nanoparticles, this review focuses on the recent development and application of nanoparticles in the early diagnosis and treatment of the three common cancers (lung cancer, liver cancer, and breast cancer) by using quantum dots, magnetic nanoparticles, and gold nanoparticles.

## 1. Introduction

**Figure 1:**
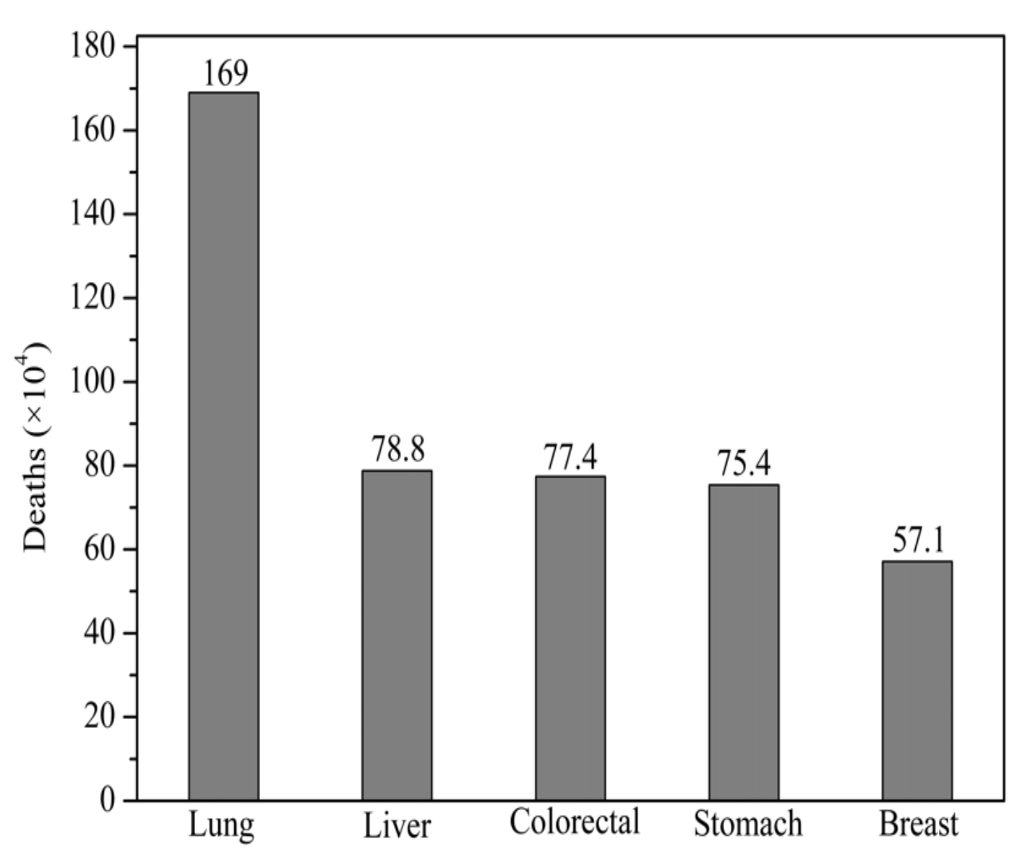
The top five ranking of the most common cancer deaths across the world from the World Health Organization’s report in 2015 [1].

Cancer is a malignant tumor with a high mortality rate, and many people die from various types of cancers every year in the world. In 2015, the World Health Organization report ranked the top five most common cancer deaths across the world, including lung cancer, liver cancer, colorectal cancer, stomach cancer, and breast cancer (Figure 1). In 2015, 1.69 million people died of lung cancer in the world. Breast cancer is more common in women, and 571,000 people, including men, died of breast cancer in 2015. Furthermore, lung cancer, liver cancer, and breast cancer are highly fatal and easily overlooked.

Based on the differences in growth and spread of cancer cells, lung cancers are divided into small cell lung cancer and non-small cell lung cancer. Breast cancer is a serious malignant tumor that threatens the health of women [2], and there are more than 6 million breast cancer patients worldwide currently. Liver cancers consist of primary liver cancer and secondary liver cancer, which are highly aggressive. The treatment of these cancers puts patients under tremendous financial and psychological stress, and early diagnosis can help patients recover quicker and ease their burdens. 

For these cancers, diagnostic techniques mainly include computed tomography (CT), magnetic resonance imaging (MRI), positron emission computed tomography (PET), color Doppler ultrasound imaging (CDI), and biochemical indicators, but their poor sensitivity and accuracy limits their use in early diagnosis of cancer. For instance, PET relies on an efficient radioactive substance, and testing costs are very high, which hinder its wide use. PET/CT has relatively high specificity and accuracy for lung cancer imaging compared with PET alone, and has additional value in detecting extrathoracic metastasis, such as metastasis in the liver [3,4]. Barabasch et al. investigated the accuracy of diffusion-weighted magnetic resonance imaging (DWI-MRI) and PET/CT, and concluded that DWI-MRI appeared to be superior to PET/CT for early assessment of treatment response in patients with hepatic metastases of common solid tumors. DWI-MRI accurately predicts response before changes of tumor size or contrast enhancement are observable on contemporary cross-sectional imaging, such as contrast-enhanced MRI or CT. It may therefore be useful to guide treatment decisions in patients undergoing lobar Y90-radioembolization [5]. Concurrently, cancer therapy can be approximately segmented into two categories: surgery and non-surgery. Surgical excision is limited by the size of tumors and the invasion of metastasis to adjacent tissues and organs [6]. Non-surgical treatment of cancers mainly includes radiotherapy and chemotherapy. Many systemic and genotoxic chemotherapies have debilitating side effects, including chemoresistance, and also induce cellular senescence in normal tissues [7,8]. Thus, there are still many challenges in the early diagnosis and treatment of cancers.

Challenges often mean new opportunities, especially with the advent of nanomedicine. A novel aptameric nanobiosensor based on a self-assembled DNA-MoS_2_ nanosheet architecture shows high sensitivity and rapid response for biomolecule detection [9]. Nanofluidics is a strong candidate for sample processing, especially for trace samples [10]. The nanoparticles applied in the early diagnosis and treatment of cancers are also an integral part of nanomedicine.

Nanoparticles refer to particles with a size between 1 and 100 nm, both for atypical microscopic and macroscopic systems, and some nanoparticles, such as quantum dots, magnetic nanoparticles, and gold nanoparticles, are widely used in biomedicine fields. Quantum dots are very small semiconductor particles; many types of quantum dots emit light of specific frequencies if electricity or light is applied to them, and these frequencies can be precisely tuned by changing the dots’ size. Magnetic nanoparticles have showed a promise in biomedical applications, such as magnetic hyperthermia and photothermal bimodal treatment [11], enhancing MRI, and improving the delivery efficiency of drugs [12]. As a noble metal, gold nanoparticles promote the scattering and absorption of light at resonant wavelengths [13]. This property can improve our understanding of the short range dependence of the adsorbate-induced localized surface plasmon resonance peak shift on nanoparticle structure and composition reported here, which will translate to significant improvements in the sensitivity of refractive-index-based nanoparticle nanosensors [14] and easy surface modification for improving the diagnosis and treatment of cancer. Owing to the amenability of the synthesis and functionalization of gold nanoparticles, their exploitation is prevalent in medicine [15]. Additionally, dispersion or aggregation of gold nanoparticles will display different colors, which is convenient for a readout without any specialized equipment [16]. However, there is no comprehensive overview of the application of nanoparticles in the early diagnosis and treatment of the three common cancers. Therefore, this review focuses on the most recent development and application of nanoparticles, mainly quantum dots, magnetic nanoparticles, and gold nanoparticles, in the early diagnosis and treatment of lung cancer, liver cancer, and breast cancer.

## 2. Strategies with Nanoparticles for Early Diagnosis of the Three Common Cancers

Over the years, the increasing number of cancer patients worldwide has put tremendous economic pressure on both countries and individuals. Less-developed countries currently account for about 57% of cases and 65% of cancer deaths in the world [17]. Therefore, except for the active and necessary prevention, early diagnosis is also very crucial. In order to improve the early diagnosis of lung cancer, liver cancer, and breast cancer, some new advances have been made using the unique properties of quantum dots, magnetic nanoparticles, and gold nanoparticles.

### 2.1. Detecting Cancer Biomarkers

Cancer biomarkers refer to substances produced directly by tumor cells or induced by tumor cells from non-tumor cells. Through the detection of these biomarkers, the presence and prognosis of the tumor can be judged (Table 1).

#### 2.1.1. Nanoprobe and Biosensor

A nanoprobe is an optical device for spotting very small substances that can respond to the presence of chemical species or monitor physical parameters and could be used for the non-invasive diagnosis of cancer. Quantum dots are common nanoprobes with several outstanding features, such as quantum confinement effects, an extremely narrow emission spectrum, high brightness, and good resistance to fluorescence quenching [26,27]. The next-generation nanoprobes are supposed to be stable, highly specific, and ultrasmall in early cancer diagnostic platforms [28]. A nanoprobe engineered through oriented conjugation of quantum dots with single-domain antibodies showed an advantage for cancer biomarkers detection, and it had a hydrodynamic diameter below 12 nm [29]. By quantum dot-tagged cancer cells and multifunctional quantum dot probes, Bertolini et al. achieved sensitive and multicolor fluorescence imaging of cancer cells in vivo [30]. Besides this, Yao et al. fabricated magnetofluorescent carbon quantum dots (MFCQDs) using a combination of waste crab shell and transition-metal ions Gd^3+^, Mn^2+^, and Eu^3+^ as potential diagnostic probes, from which the chitin acted not only as a carbon source but also as a chelating ligand to form complexes with transition-metal ions [31]. For safety and environmental protection, it is essential to reduce toxic heavy metal use for quantum dots [32]. When a small part of the quantum dots does not accumulate at the tumor site, the membrane-impermeable etchant can rapidly quench the quantum dots and acquire highly tumor-specific signals through cation exchange in vivo [33]. Furthermore, near-infrared ray (NIR) bioprobes may be applied, and the optimal NIR bioprobes must possess emission color tenability, a large Stokes shift, high photo-stability, and multimodal detection [34].

A biosensor is an instrument that is sensitive to biological substances and converts their concentrations into electrical signals for detection. Gold nanoparticles equipped with effective fluorescence quenching can detect specific molecular biomarkers. DNA biosensors based on gold-nanoparticles-modified graphene oxide were constructed to detect two biomarkers, human epidermal growth factor receptor-2 (HER2) and the cell surface protein CD24, of breast cancer. Using amperometric detection of a horse radish peroxidase label, detection limits of 0.16 and 0.23 nM were obtained with a sensitivity of 378 and 219 nA/nM for HER2 and CD24, respectively [35]. In addition, electrogenerated chemiluminescence detection of DNA with gold nanoparticles may also be a selected strategy to improve the diagnosis of cancer [36]. Recently, it was reported that Castro-Beltrán et al. sought out a way to perfect frequency comb construction through adding gold nanoparticles to the microlaser element, which took up less space and required about 1000 times less power than current comb technology [37]. This biosensor shows promise to become a biochemical detector for the early diagnosis of cancer.

#### 2.1.2. Microarray

A microarray is a convoluted lab-on-a-chip for sample detection, and it is usually built on a solid substrate (glass slide, silicon slide). In general, types of microarrays include DNA microarrays, protein microarrays, peptide microarrays, and antibody microarrays. α-1-microglobulin/bikunin precursor (AMBP), peroxiredoxin 2 (PRDX2), and Parkinson’s disease protein 7 (PARK7) are three protein markers associated with lung cancers. Bilan et al. researched a quantum-dot-based suspension microarray for multiplexed analysis of the three proteins with an obvious distinction between the control and clinical samples [38]. Circulating microRNAs may be potential noninvasive biomarkers for cancer detection. In clinical trials, Li et al. utilized a nano-quantum dots microarray to test serum samples from non-small cell lung cancer patients, and found that some serum miRNA were significantly different from the control, such as smiR-16-5p and miR-17b-5p [39].

#### 2.1.3. Quantum Dots Enhancing Technique

Trastuzumab is a humanized monoclonal antibody against HER2 expression in breast cancer patients. Immunohistochemistry with 3,3′-diaminobenzidine (IHC-DAB) is applied to analyse the enzymatic activity of horseradish peroxidase, but it cannot be quantitative. Hence, a developed immunohistochemistry (IHC) technique with quantum dots-conjugated trastuzumab using single-particle imaging has been discovered to measure the HER2 expression [40]. Hybridization chain reaction (HCR) is a desirable method for signal amplification, and a simple quantum dots-Ru complex assembling dyads based on HCR showed higher sensitivity in cancer cell detection and cellular imaging and provided more reliable test results [41].

### 2.2. Tissue Imaging

Clear depictions of tumor boundaries allow for accurate judgments of tumor distribution and its response to surgical removal and adjuvant therapies [42]. Nowadays, MRI is a powerful tumor imaging modality of liver cancer diagnosis, but its sensitivity is still lacking when applied to molecule and cell imaging for early detection. Therefore, the development of early diagnosis methods for liver cancer is critical. As is known to many, magnetic nanoparticles can create microscopic field gradients in a strong magnetic field, thereby shortening the longitudinal (*T*_1_) or transverse relaxation times (*T*_2_) of nearby nuclei and highlighting the areas [43]. Magnetic nanoparticles, particularly iron-oxide nanoparticles, can be used in liver imaging and cell tracking. Glycosaminoglycan-targeted superparamagnetic iron-oxide nanoparticles (SPIONs) can be applied in MRI to track human hepatocellular liver carcinoma (HepG2) cells [44]. A multifunctional fluorescent magnetic nanoparticles matrix could identify lung cancer stem cells specifically and be used for tumor xenograft imaging, which is a cancer diagnostic agent that functions through modifying diverse fluorescence dyes and targeting ligands [45]. Moreover, taking advantage of a cancer-targeting ligand folate and NIR fluorescent dye Cy5.5, Yang et al. built cross-linked magnetic nanoparticles for cancer-targeted dual optical/magnetic resonance imaging with good biocompatibility and biodegradability. The results in vitro and in vivo demonstrated the potential utility of Cy5.5- and folate-conjugated magnetic nanoparticles as dual-imaging probes for specific cancer-targeted MR/NIR imaging applications [46]. Multimodal imaging methods provide an efficient way to overcome the weakness of a single imaging modality [47].

## 3. Multiple Ways with Nanoparticles for Treatment of the Three Common Cancers

In real organ cancer, surgical resection is common, but not necessarily the best solution, and it is needed to prevent the spread and metastasis of cancer cells. The tumor microenvironment is attracting more and more researchers’ attention in recent years, and it is distinct from normal tissues. For example, hypoxia [48], the blocking of lymphatic drainage [49], and tumor-infiltrating lymphocytes production give tumor tissues unique physical and chemical properties [50,51]. Targeted nanoparticles based on the unique physicochemical properties of the tumor microenvironment have been extensively researched for the therapy of cancers.

### 3.1. Inhibition of Cancer Migration by Increasing the Nuclear Membrane Stiffness of Cancer Cells

As a tumor grows, cancer cells are often able to metastasize and spread to nearby tissues and even distant organs, such as the lungs, liver, and brain [52], It is worthwhile to slow cancer cell migration and invasion, because nuclear stiffness of the cell can decrease cell migration to a large extent. Gold nanoparticles were modified with three ligands containing methoxy-polyethylene glycol thiol (PEG), RGD (RGDRGDRGDRGDPGC) peptides, and nuclear localization signal (NLS, CGGGPKKKRKVGG) peptides, and the targeting gold nanoparticles were absorbed by the nuclear membrane of the cancer cell and stimulated the overexpression of lamin A/C proteins as well. As a result, the nuclear stiffness of the cancer cell was increased and it could not effectively spread [53]. In addition, further investigation of binding kinetics with the cell membrane is ponderable. Thus, effectively restricting the spread of cancer cells deserves our attention because it will give doctors and patients more time to fight.

### 3.2. Immunotherapy

In patients with tumors, it is difficult to produce an effective immune response for their reduced immunogenicity. Vaccines have great potential in cancer immunotherapy because of their ability to cause a specific, strong, and persistent immune response, but a fatal drawback of cancer vaccines is their low immune-stimulating capacity [54]. Gold nanoparticles can be a good vaccine vector due to their suitable payload [55], low immunogenicity, and biocompatibility. Simultaneously, for gold nanoparticle-based vaccines, the size is a critical factor for inducing immune responses. In order to discover the optimal size, a series of studies were conducted, such as delivery efficiency, restimulation assay, and tumor-prevention studies. Eventually, their results suggested that the size threshold of gold nanoparticle-based vaccines was between 10 and 22 nm [56]. In addition, immunotherapy using gold nanoparticles can be an adjuvant treatment to surgery [57].

Dendritic cells are a class of antigen-presenting cells with high efficiency, and suppressor of cytokine signaling 2 (SOCS2) was found to be a critical factor for tumor-immune surveillance in dendritic cells [58]. Besides this, a high-density peptide gold nanoparticle can stimulate cytotoxic T lymphocytes better than free peptides and dendritic cells uptake with minimal toxicity [59]. Adoptive immunotherapy (AIT) is also a strategy for cancer treatment, and the enrichment and expansion of tumor-specific T cells has progressed with an iron-Dextran nanoparticle [60]. This also gives insight into other cancers.

### 3.3. Photothermal Therapy and Magnetic Hyperthermia

As is known to many, light/magnet-induced heat, such as photothermal therapy (PTT) and magnetic hyperthermia, is a way to treat cancer. Triple-negative breast cancer is a kind of breast cancer without the routinely targeted ER, PR, and HER2 expression, and it represents an important clinical challenge because these cancers do not respond to endocrine therapy or other available targeted agents [61]. In view of our knowledge and understanding of the tumor microenvironment, Sethi et al. have structured a three-dimensional (3D) co-culture system which consists of mCherry fluorescent-protein-expressing 4T1 tumor cells, green fluorescence protein (GFP)-expressing C166 endothelial cells, and murine embryonic fibroblasts [62]. With the existence of an alternating magnetic field, a high concentration of magnetic nanoparticles (about 1 mg/mL) showed enhanced cell killing, indicating the potential in thermal therapy for secondary lung malignancies [63]. Epidermal growth factor receptor (EGFR) overexpression is a general phenomenon in non-small cell lung cancer patients, and it is convenient to utilize nanoparticles-containing inhalable aerosols for lung cancer therapy through the gas exchange of the lungs [64]. In order to address inadequate delivery and non-targeted delivery, EGFR-targeted and inhalable SPIONs for the magnetic hyperthermia of non-small cell lung cancer have been prepared. The results suggested that EGFR targeting strengthened tumor retention of SPIONs in the lung. Further, magnetic hyperthermia treatment using targeted SPIONs led to significant inhibition of lung tumor growth in vivo [65].

PTT is a way of treating various kinds of cancer, which mainly relies on the principle of converting light energy into thermal energy. The liquid exfoliation method was used to produce antimonene quantum dots, which were then modified with PEG. They showed a photothermal conversion efficacy of 45.5%, and demonstrated notable NIR-induced tumor ablation ability in MCF-7 tumor-bearing mice [18]. Magnetofluorescent carbon quantum dots have also attracted significant attention in biomedical studies due to their major role in cancer photothermal therapeutics.

### 3.4. Synergistic Therapy

Quantum dots have a wide range of applications because of their exceptional optical and electronic properties, and further surface functionalization is necessary for the treatment of cancer [66]. Zhang and Pan et al. synthesized magnetofluorescent carbon quantum dots such as the Fe-crosslinked chitosan complex FeN@CQDs with intrinsic photoluminescent and magnetic properties, conjugated with folic acid and riboflavin for targeting and PTT, and doxorubicin was further incorporated into the system to enable targeted drug delivery. The data suggested that the system can deliver the anti-cancer drug doxorubicin to target cells, release them intracellularly upon NIR irradiation, and effectively eliminate tumors through a chemo-photo synergistic therapeutic effect in a malignant liver tumor model with female nude mice [67]. In addition, quantum dots are an ideal cancer surgeon’s latest tool because of their relatively slow migration and their ability to maintain fluorescence for longer [68].

**Figure 2:**
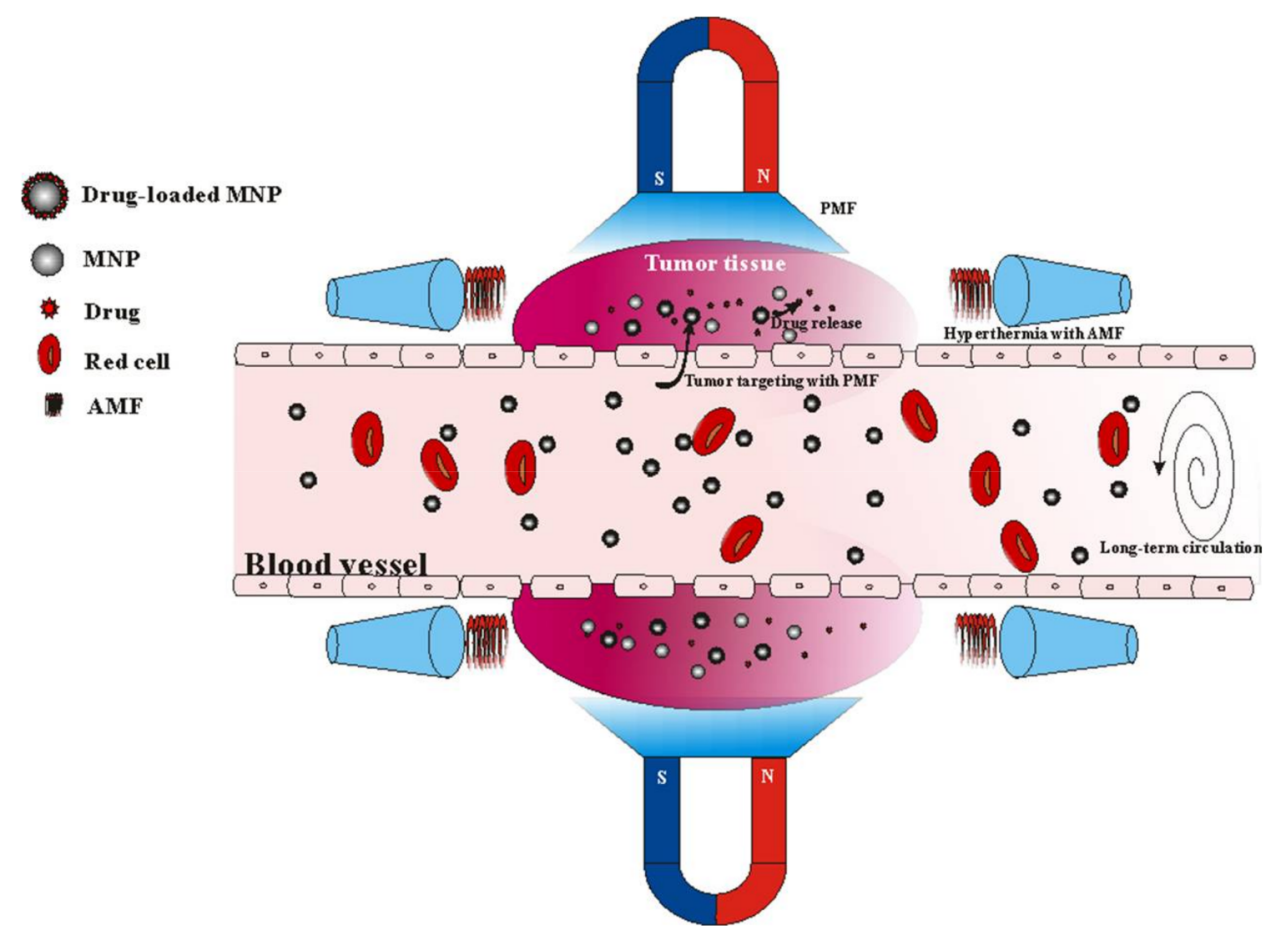
Schematic diagrams of a drug-loaded magnetic nanoparticle (MNP) with tumor targeting, hyperthermia, and long-term circulation. AMF: alternating magnetic field; PMF: permanent magnetic field.

When magnetic nanoparticles are put inside a tumor and the whole patient is placed in an alternating magnetic field, the tumor temperature will rise. The elevation of the temperature may enhance tumor oxygenation and radio- and chemosensitivity, which hopefully shrinks tumors. In the presence of alternating electromagnetic fields, iron oxide-based magnetic nanoparticles produce heat by Brownian and Néel relaxation [69]. In order to achieve the dual effect of drug and hyperthermia, one first uses a permanent magnetic field for tumor targeting and drug release, then uses an alternating magnetic field for hyperthermia (Figure 2). Besides this, nanoparticles with a hydrodynamic diameter (approximately 20–200 nm) can be applied for long-term circulation [70].

By contrast, gold nanoparticles in treatment are mainly relied on for their easy surface modification and photothermal conversion efficacy, and gold nanoparticles in diagnosis usually use their excellent optical performance. Gold nanoparticles have been widely used for the delivery of photosensitizers for the photodynamic therapy (PDT) of cancer [71].

### 3.5. Potential Combination with Nanoparticles of Cancer Treatment

In contrast to conventional cytotoxic chemotherapy, targeted cancer therapies possess specific anticancer effects and less toxicity. In general, the drug is designed to attack a certain molecular agent or pathway involved in the development of cancer [72]. Targeted drugs can achieve the following goals: block or turn off chemical signals, stop making new blood vessels, and trigger the body’s immune system [73]. Some drugs have been approved by the U.S. Food and Drug Administration for cancer-targeted therapy (Table 2), but they are used with other treatments, such as surgery and chemotherapy in most cases. Some drugs are not well-targeted, for instance, Nivolumab (Opdivo^®^) can treat colorectal cancer, non-small cell lung cancer, and renal cell carcinoma. On account of a controlled release [74] improving the stability of drug molecules and increasing drug solubility in vivo [75], nanoparticle-based carriers have obvious advantages for targeted cancer therapy. Combined with the pharmacological effects of drugs and the advantages of nanoparticles-based carriers, it would make sense to slow the development of drug resistance [76]. Moreover, the chemical elements, molar mass, and 3D models of these drugs provide ideas for the design of new drugs and better nanosystems.

Gene mutations are closely linked to the occurrence of cancer. As a crucial tumor suppressor, p53 controls cell cycle checkpoints and allows cells to repair damaged DNA or induce an apoptosis gene, and many tumors are related to their mutations [78]. Importantly, the 2017 Nobel Prize winners in physiology or medicine have found that the *period* gene, *timeless* gene, and *doubletime* gene play a significant role in the regulation of circadian rhythms, and interference with the relevant genes may contribute to the treatment of cancer.

## 4. Theranostic Nanoplatforms of the Three Common Cancers

In clinical practice, diagnosis and treatment are usually separated. However, the integration of diagnosis and treatment, which is called a theranostic, may be useful for cancer. Theranostics are specific targeted therapies based on targeted diagnostic tests. By designing and synthesizing specific nanomaterials with nanoparticles, theranostics can not only be used for the early diagnosis of cancer and simultaneous treatment, but also for monitoring the efficacy of the treatment process and adjusting it over time to achieve better treatment and a reduction in side-effects [79]. Furthermore, theranostics will have great prospects in personalized medicine in terms of factors such as age, gender, and severity of disease for better diagnostic and therapeutic effects [80].

### 4.1. Functionalized Superparamagnetic Iron Oxide

Magnetic nanoparticles have many forms, of which SPIONs is a typical representative. SPIONs are constituted of an iron-oxide core and coated with a layer of stable compounds [81]. Magnetic-resonance-guided focused ultrasound surgery (MRgFUS) can provide real-time and three-dimensional imaging, but with a low efficiency of ultrasonic energy deposition and relative insensitivity of MRI at the present stage. Wang et al. developed a nanosized theranostic superparamagnetic iron oxide nanoplatform for a clinical MRgFUS system. These SPIONs coated with PEG were further decorated with high affinity antiEGFR monoclonal antibody (Cetuximab). The results confirmed that it can improve the MRI contrast at the tumor site and enhance ultrasonic energy deposition in MRgFUS in nude rat models bearing H460 lung cancer xenografts [23]. Moreover, 54 peptide-functionalized poly (lactic-*co*-glycolic acid)-grafted dextran micelles have been synthesized, which were encapsulated by the model anti-tumor drug doxorubicin and SPIO. According to the data, it was found that the anti-tumor drug can be targeted for delivery to liver cancer cells, and compared with the same drug without SPIO, the drug efficacy is significantly enhanced and with better MRI [82]. Besides this, hyaluronan (HA)-SPIONs exhibited an exquisite ability for targeted MRI and to have highly effective PTT induced by near-infrared laser irradiation for CD44 HA receptors highly expressing breast cancer in both in vitro and in vivo studies [19]. As magnetic nanoparticles, they can be degraded to Fe ions in the body, which mitigates the potential toxicity [83].

To prepare antibody-conjugated nanoparticles, controllability, stability, and reliability need to be considered [84]. The anticancer drug trastuzumab (Herceptin^®^) is a kind of monoclonal antibody that targets a certain protein, HER2, which is located in the surface of some cancer cells. An iron-oxide nanoparticle and doxorubicin-loaded nanocarrier with trastuzumab has been prepared for studying its imaging and treatment of cancer. Based on the results of cellular uptake in vitro and in a tumor xenograft model with SK-BR-3 cells in vivo, it illuminated the potential for early stage cancer diagnosis and simultaneous therapy [21]. A suitable molecular mass and average size of monoclonal antibodies need to be considered when designing nanosystems. In addition, a conceptual nanoparticle with a silica-coated iron-oxide magnetic core was also designed as a valuable theranostic for diagnostic imaging and drug delivery systems [85].

### 4.2. Gold Nanoparticles as a Theranostic Application

Gold nanoparticles have been extensively used in medicine due their unique properties, such as easy surface modification, photothermal conversion effect, and high X-ray absorption coefficient [86,87]. With their high X-ray absorption coefficient, gold nanoparticles have been used as an imaging agent or a radiosensitizer for the diagnosis of cancer [88,89].

**Scheme 1:**
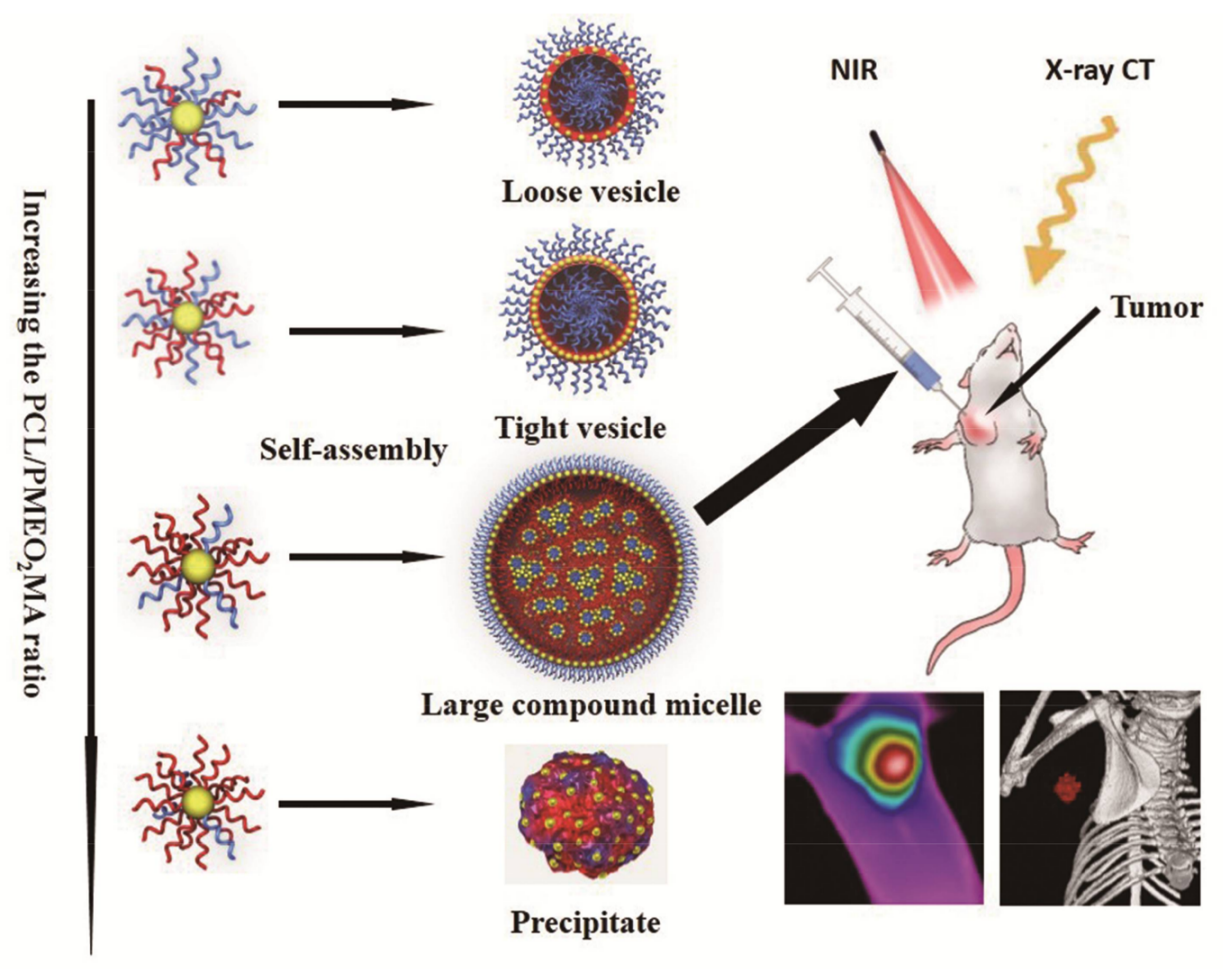
Schematic representation of assemblies composed by poly (ε-caprolactone) and hydrophilic poly 2-(2-methoxyethoxy) ethyl methacrylate-grafted Au nanoparticles (AuNPs) and their potential application in photothermal therapy and computed tomography (CT) imaging of cancer. Reproduced with permission [92], Ivyspring International Publisher, 2014. NIR: near infrared.

After absorbing energy, part of the energy is emitted as scattered light for imaging, and the remaining energy is attenuated in a nonradiative form and converted to heat as a target ablation of the tumor tissue, which is the mechanism of gold nanoparticles used in a theranostic [90]. The size of gold nanoparticles usually affects the CT contrast and radiosensitization effects, so adjusting their sizes is a requirement. Dou et al. found that gold nanoparticles with a size of ~13 nm could simultaneously possess superior CT contrast ability and significant radioactive disruption, and the Monte Carlo method indicated that the inhomogeneity of gold atom distributions caused by sizes may influence secondary ionization in whole X-ray interactions, which may serve as multifunctional adjuvants for a clinical X-ray theranostic application [91]. Besides this, Deng and Zhang et al. used 14-nm gold nanoparticles as building blocks for self-assembly, which were anchored by thiolated hydrophobic poly (ε-caprolactone) (PCL) and hydrophilic poly 2-(2-methoxyethoxy) ethyl methacrylate (PMEO_2_MA), showing the possibility for their use as theranostic agents (Scheme 1) [92].

In addition, a gold nanoparticle probe has been developed and evaluated for human telomeres. The results indicated that the average diameter of telomeres was about 180 nm [93], and it is possible to investigate the telomeres of cancer cells for understanding basic biological processes and searching for more efficient methods for the early diagnosis and treatment of cancers. Besides this, photo-medical techniques with NIR excitation sources can be achieved for cancer theranostics [94].

## 5. Conclusions

At present, many breakthroughs have been achieved in the development of nanoparticles for the early diagnosis and treatment of cancers. Tumor-associated antigens and tumor-specific antigens have been further researched and developed for cancer prevention [95]. However, most of these studies are just in their infancy, and focus on different cancer cell lines and xenograft models. For tumor targeting and imaging of nanoparticles, their clearance pathways mainly contain renal clearance and a hepatic route that is relative to the size, shape, and surface properties of the nanoparticles [96]. Simultaneously, nanoparticles can improve the early diagnosis and treatment of cancer, but it is crucial to understand how the nanoparticles distribute in tumors, are removed from normal tissues, and are eventually excreted from the body. Finally, it may be a viable approach to design and utilize novel nanoparticles as anticancer weapon in the future through molecular biological techniques, nanomedicine, bioinformatics principles, and big data science. For example, a nanosensor chip could be located within a cell phone case, and it is thought that a person would just breathe on the cell phone’s housing. The nanosensor could be used as a completely non-invasive diagnosis and measurement method. On the basis of the correlation between nitrous oxide and lung cancer, scientists have already trained dogs to accomplish this diagnostic task, but the nanosensor possesses a special “nose” for “sniffing out cancer”. Scientists and technologists believe that the health applications are endless with this new nanosensor.

## Figures and Tables

**Table 1:** Common cell lines of the three cancers from preclinical research.

Cancer	Cell Lines	Biomarkers	Tumorigenic	Nanoplatform	Refrence
Breast	MCF-7	ER, PR	Nude mice	QDs	[18]
	MDA-MB-231	CD44 HA receptor	Nude mice	SPIONs	[19]
	T-47D	ER, PR	Nude mice	-	[20]
	SK-BR-3	HER2	Nude mice	IONPs	[21]
Lung	A549	CD90, HPRT	Nude mice	-	[22]
	NCI-H460	HPRT	Nude mice	IONPs	[23]
	SK-MES-1	CD24	Nude mice	-	[24]
Liver	Hep G2	CD90, CD44	-	-	-
	Huh7	CD133	Mice	-	[25]

ER, estrogen receptor; PR, progesterone receptor; HA, hyaluronan; HPRT, hypoxanthine-guanine phosphoribosyltransferase; IONPs, iron-oxide nanoparticles; SPIONs, superparamagnetic iron-oxide nanoparticles; QDs, quantum dots.

**Table 2:** Targeted drug therapies that have been approved by the U.S. Food and Drug Administration. Data from the National Cancer Institute [77].

Cancer	Targeted Drug Therapies	Molar Mass (g/mol)	Chemical Formula	Target
Breast	Everolimus (Afinitor^®^)	958.224	C_53_H_83_NO_14_	-
	Toremifene (Fareston^®^)	405.959	C_26_H_28_ClNO	-
	Trastuzumab (Herceptin^®^) *	145,531.5	C_6470_H_10012_N_1726_O_2013_S_42_	HER2/neu
	Anastrozole (Arimidex^®^)	293.366	C_17_H_19_N_5_	-
	Lapatinib (Tykerb^®^)	581.058	C_29_H_26_ClFN_4_O_4_S	-
Lung	Bevacizumab (Avastin^®^) *	149,196.82	C_6638_H_10160_N_1720_O_2108_S_44_	VEGF-A
	Crizotinib (Xalkori^®^)	450.337	C_21_H_22_Cl_2_FN_5_O	-
	Erlotinib (Tarceva^®^)	393.436	C_22_H_23_N_3_O_4_	-
	Nivolumab (Opdivo^®^) *	143,600	C_6362_H_9862_N_1712_O_1995_S_42_	PD-1
Liver	Sorafenib (Nexavar^®^)	464.825	C_21_H_16_ClF_3_N_4_O_3_	-
	Regorafenib (Stivarga^®^)	482.82	C_21_H_17_ClF_4_N_4_O_4_	-

* Monoclonal antibody.

## References

[1] World Health Organization (2017). Cancer Fact Sheet. http://www.who.int/mediacentre/factsheets/fs297/en/.

[2] Miller K.D., Siegel R.L., Lin C.C., Mariotto A.B., Kramer J.L., Rowland J.H., Stein K.D., Alteri R., Jemal A. (2016). Cancer treatment and survivorship statistics, 2016. CA A Cancer J. Clin..

[3] Zhu A., Lee D., Shim H. (2011). Metabolic positron emission tomography imaging in cancer detection and therapy response. Seminars in Oncology.

[4] Liu N., Ma L., Zhou W., Pang Q., Hu M., Shi F., Fu Z., Li M., Yang G., Yu J. (2010). Bone metastasis in patients with non-small cell lung cancer: The diagnostic role of f-18 fdg pet/ct. Eur. J. Radiol..

[5] Barabasch A., Kraemer N.A., Ciritsis A., Hansen N.L., Lierfeld M., Heinzel A., Trautwein C., Neumann U., Kuhl C.K. (2015). Diagnostic accuracy of diffusion-weighted magnetic resonance imaging versus positron emission tomography/computed tomography for early response assessment of liver metastases to y90-radioembolization. Investig. Radiol..

[6] Martin T.A., Ye L., Sanders A.J., Lane J., Jiang W.G. (2013). Cancer Invasion and Metastasis: Molecular and Cellular Perspective.

[7] Demaria M., O’Leary M.N., Chang J., Shao L., Liu S., Alimirah F., Koenig K., Le C., Mitin N., Deal A.M. (2017). Cellular senescence promotes adverse effects of chemotherapy and cancer relapse. Cancer Discov..

[8] Jackson S.P., Bartek J. (2009). The DNA-damage response in human biology and disease. Nature.

[9] Ge J., Ou E.-C., Yu R.-Q., Chu X. (2014). A novel aptameric nanobiosensor based on the self-assembled DNA–mos 2 nanosheet architecture for biomolecule detection. J. Mater. Chem. B.

[10] Koltonow A.R., Huang J. (2016). Two-dimensional nanofluidics. Science.

[11] Espinosa A., Di Corato R., Kolosnjaj-Tabi J., Flaud P., Pellegrino T., Wilhelm C. (2016). Duality of iron oxide nanoparticles in cancer therapy: Amplification of heating efficiency by magnetic hyperthermia and photothermal bimodal treatment. ACS Nano.

[12] Williams H.M. (2017). The application of magnetic nanoparticles in the treatment and monitoring of cancer and infectious diseases. Biosci. Horiz. Int. J. Stud. Res..

[13] Fratoddi I. (2017). Hydrophobic and hydrophilic Au and Ag nanoparticles. Breakthroughs and perspectives. Nanomaterials.

[14] Haes A.J., Zou S., Schatz G.C., Van Duyne R.P. (2004). Nanoscale optical biosensor: Short range distance dependence of the localized surface plasmon resonance of noble metal nanoparticles. J. Phys. Chem. B.

[15] Tiwari P.M., Vig K., Dennis V.A., Singh S.R. (2011). Functionalized gold nanoparticles and their biomedical applications. Nanomaterials.

[16] Young K.L., Ross M.B., Blaber M.G., Rycenga M., Jones M.R., Zhang C., Senesi A.J., Lee B., Schatz G.C., Mirkin C.A. (2014). Using DNA to design plasmonic metamaterials with tunable optical properties. Adv. Mater..

[17] Torre L.A., Bray F., Siegel R.L., Ferlay J., Lortet-Tieulent J., Jemal A. (2015). Global cancer statistics, 2012. CA A Cancer J. Clin..

[18] Tao W., Ji X., Xu X., Islam M.A., Li Z., Chen S., Saw P.E., Zhang H., Bharwani Z., Guo Z. (2017). Antimonene quantum dots: Synthesis and application as near-infrared photothermal agents for effective cancer therapy. Angew. Chem. Int. Ed. Engl..

[19] Yang R.M., Fu C.P., Fang J.Z., Xu X.D., Wei X.H., Tang W.J., Jiang X.Q., Zhang L.M. (2017). Hyaluronan-modified superparamagnetic iron oxide nanoparticles for bimodal breast cancer imaging and photothermal therapy. Int. J. Nanomed..

[20] Yu S., Kim T., Yoo K.H., Kang K. (2017). The t47d cell line is an ideal experimental model to elucidate the progesterone-specific effects of a luminal a subtype of breast cancer. Biochem. Biophys. Res. Commun..

[21] Choi W.I., Lee J.H., Kim J.Y., Heo S.U., Jeong Y.Y., Kim Y.H., Tae G. (2015). Targeted antitumor efficacy and imaging via multifunctional nano-carrier conjugated with anti-HER2 trastuzumab. Nanomedicine.

[22] Kellar A., Egan C., Morris D. (2015). Preclinical murine models for lung cancer: Clinical trial applications. BioMed. Res. Int..

[23] Wang Z., Qiao R., Tang N., Lu Z., Wang H., Zhang Z., Xue X., Huang Z., Zhang S., Zhang G. (2017). Active targeting theranostic iron oxide nanoparticles for mri and magnetic resonance-guided focused ultrasound ablation of lung cancer. Biomaterials.

[24] Pal H.C., Sharma S., Strickland L.R., Agarwal J., Athar M., Elmets C.A., Afaq F. (2013). Delphinidin reduces cell proliferation and induces apoptosis of non-small-cell lung cancer cells by targeting EGFR/VEGFR2 signaling pathways. PLoS ONE.

[25] Yang W., Wang C., Lin Y., Liu Q., Yu L.X., Tang L., Yan H.X., Fu J., Chen Y., Zhang H.L. (2012). Ov6^+^ tumor-initiating cells contribute to tumor progression and invasion in human hepatocellular carcinoma. J. Hepatol..

[26] Wegner K.D., Hildebrandt N. (2015). Quantum dots: Bright and versatile in vitro and in vivo fluorescenceimaging biosensors. Chem. Soc. Rev..

[27] Lim S.J., Zahid M.U., Le P., Ma L., Entenberg D., Harney A.S., Condeelis J., Smith A.M. (2015). Brightness-equalized quantum dots. Nat. Commun..

[28] Nabiev I. (2017). Quantum Dot-Based Hybrid Nanostructures and Energy Transfer on the Nanoscale for Single- and Multi-Photon Imaging and Cancer Diagnostics.

[29] Sukhanova A., Millot J.-M., Pluot M., Cohen J.H., Nabiev I. Diagnostic Nanoprobes Based on the Conjugates of Quantum Dots and Single-Domain Antibodies for Cancer Biomarkers Detection in Immunohistochemistry and Flow Cytometry. Proceedings of the 2015 International Conference on Biomedical Engineering and Computational Technologies (SIBIRCON).

[30] Bertolini G., Paleari L., Catassi A., Roz L., Cesario A., Sozzi G., Russo P. (2008). In vivo cancer imaging with semiconductor quantum dots. Curr. Pharm. Anal..

[31] Yao Y.Y., Gedda G., Girma W.M., Yen C.L., Ling Y.C., Chang J.Y. (2017). Magnetofluorescent carbon dots derived from crab shell for targeted dual-modality bioimaging and drug delivery. ACS Appl. Mater. Interfaces.

[32] Maxwell T., Banu T., Price E., Tharkur J., Campos M.G., Gesquiere A., Santra S. (2015). Non-cytotoxic quantum dot-chitosan nanogel biosensing probe for potential cancer targeting agent. Nanomaterials.

[33] Liu X., Braun G.B., Qin M., Ruoslahti E., Sugahara K.N. (2017). In vivo cation exchange in quantum dots for tumor-specific imaging. Nat. Commun..

[34] Liu T.-M., Conde J., Lipiński T., Bednarkiewicz A., Huang C.-C. (2016). Revisiting the classification of NIR-absorbing/emitting nanomaterials for in vivo bioapplications. NPG Asia Mater..

[35] Saeed A.A., Sanchez J.L.A., O’Sullivan C.K., Abbas M.N. (2017). DNA biosensors based on gold nanoparticles-modified graphene oxide for the detection of breast cancer biomarkers for early diagnosis. Bioelectrochemistry.

[36] Wang X., Ge L., Yu Y., Dong S., Li F. (2015). Highly sensitive electrogenerated chemiluminescence biosensor based on hybridization chain reaction and amplification of gold nanoparticles for DNA detection. Sens. Actuators B Chem..

[37] Castro-Beltrán R., Diep V.M., Soltani S., Gungor E., Armani A.M. (2017). Plasmonically enhanced Kerr frequency combs. ACS Photon..

[38] Bilan R., Ametzazurra A., Brazhnik K., Escorza S., Fernandez D., Uribarri M., Nabiev I., Sukhanova A. (2017). Quantum-dot-based suspension microarray for multiplex detection of lung cancer markers: Preclinical validation and comparison with the luminex xMAP**^®^** system. Sci. Rep..

[39] Fan L., Qi H., Teng J., Su B., Chen H., Wang C., Xia Q. (2016). Identification of serum mirnas by nano-quantum dots microarray as diagnostic biomarkers for early detection of non-small cell lung cancer. Tumour Biol..

[40] Miyashita M., Gonda K., Tada H., Watanabe M., Kitamura N., Kamei T., Sasano H., Ishida T., Ohuchi N. (2016). Quantitative diagnosis of HER2 protein expressing breast cancer by single-particle quantum dot imaging. Cancer Med..

[41] Zhang Z., Xia X., Xiang X., Huang F., Han L. (2018). Quantum dots-ru complex assembling dyads for cancer cell detection and cellular imaging based on hybridization chain reaction. Sens. Actuators B Chem..

[42] Revia R.A., Zhang M. (2016). Magnetite nanoparticles for cancer diagnosis, treatment, and treatment monitoring: Recent advances. Mater. Today.

[43] Sodickson A.D., Sodickson D.K. (2016). Introductory magnetic resonance imaging physics. Handbook of Neuro-Oncology Neuroimaging.

[44] Yang R.M., Fu C.P., Li N.N., Wang L., Xu X.D., Yang D.Y., Fang J.Z., Jiang X.Q., Zhang L.M. (2014). Glycosaminoglycan-targeted iron oxide nanoparticles for magnetic resonance imaging of liver carcinoma. Mater. Sci. Eng. C Mater. Biol. Appl..

[45] Zhou X., Chen L., Wang A., Ma Y., Zhang H., Zhu Y. (2015). Multifunctional fluorescent magnetic nanoparticles for lung cancer stem cells research. Colloids Surf. B Biointerfaces.

[46] Yang H.M., Park C.W., Park S., Kim J.D. (2018). Cross-linked magnetic nanoparticles with a biocompatible amide bond for cancer-targeted dual optical/magnetic resonance imaging. Colloids Surf. B Biointerfaces.

[47] Shin T.-H., Choi Y., Kim S., Cheon J. (2015). Recent advances in magnetic nanoparticle-based multi-modal imaging. Chem. Soc. Rev..

[48] Zhang X., Ng H.L.H., Lu A., Lin C., Zhou L., Lin G., Zhang Y., Yang Z., Zhang H. (2016). Drug delivery system targeting advanced hepatocellular carcinoma: Current and future. Nanomedicine.

[49] Greish K. (2010). Enhanced permeability and retention (EPR) effect for anticancer nanomedicine drug targeting. Cancer Nanotechnology: Methods Protocols.

[50] Jain R.K., Martin J.D., Stylianopoulos T. (2014). The role of mechanical forces in tumor growth and therapy. Annu. Rev. Biomed. Eng..

[51] Spill F., Reynolds D.S., Kamm R.D., Zaman M.H. (2016). Impact of the physical microenvironment on tumor progression and metastasis. Curr. Opin. Biotechnol..

[52] Tseng L., Hsu N., Chen S., Lu Y., Lin C., Chang D., Li H., Lin Y., Chang H., Chao T. (2013). Distant metastasis in triple-negative breast cancer. Neoplasma.

[53] Ali M.R.K., Wu Y., Ghosh D., Do B.H., Chen K., Dawson M.R., Fang N., Sulchek T.A., El-Sayed M.A. (2017). Nuclear membrane-targeted gold nanoparticles inhibit cancer cell migration and invasion. ACS Nano.

[54] Rosenthal J.A., Chen L., Baker J.L., Putnam D., DeLisa M.P. (2014). Pathogen-like particles: Biomimetic vaccine carriers engineered at the nanoscale. Curr. Opin. Biotechnol..

[55] Niikura K., Matsunaga T., Suzuki T., Kobayashi S., Yamaguchi H., Orba Y., Kawaguchi A., Hasegawa H., Kajino K., Ninomiya T. (2013). Gold nanoparticles as a vaccine platform: Influence of size and shape on immunological responses in vitro and in vivo. ACS Nano.

[56] Kang S., Ahn S., Lee J., Kim J.Y., Choi M., Gujrati V., Kim H., Kim J., Shin E.C., Jon S. (2017). Effects of gold nanoparticle-based vaccine size on lymph node delivery and cytotoxic T-lymphocyte responses. J. Control. Release.

[57] Chen Q., Xu L., Liang C., Wang C., Peng R., Liu Z. (2016). Photothermal therapy with immune-adjuvant nanoparticles together with checkpoint blockade for effective cancer immunotherapy. Nat. Commun..

[58] Nirschl C.J., Suarez-Farinas M., Izar B., Prakadan S., Dannenfelser R., Tirosh I., Liu Y., Zhu Q., Devi K.S.P., Carroll S.L. (2017). Ifngamma-dependent tissue-immune homeostasis is co-opted in the tumor microenvironment. Cell.

[59] Lin A.Y., Lunsford J., Bear A.S., Young J.K., Eckels P., Luo L., Foster A.E., Drezek R.A. (2013). High-density sub-100-nm peptide-gold nanoparticle complexes improve vaccine presentation by dendritic cells in vitro. Nanoscale Res. Lett..

[60] Perica K., Bieler J.G., Schütz C., Varela J.C., Douglass J., Skora A., Chiu Y.L., Oelke M., Kinzler K., Zhou S. (2015). Enrichment and expansion with nanoscale artificial antigen presenting cells for adoptive immunotherapy. ACS Nano.

[61] Hudis C.A., Gianni L. (2011). Triple-negative breast cancer: An unmet medical need. Oncologist.

[62] Sethi P., Jyoti A., Swindell E.P., Chan R., Langner U.W., Feddock J.M., Nagarajan R., O’Halloran T.V., Upreti M. (2015). 3d tumor tissue analogs and their orthotopic implants for understanding tumor-targeting of microenvironment-responsive nanosized chemotherapy and radiation. Nanomed. Nanotechnol. Biol. Med..

[63] Stocke N.A., Sethi P., Jyoti A., Chan R., Arnold S.M., Hilt J.Z., Upreti M. (2017). Toxicity evaluation of magnetic hyperthermia induced by remote actuation of magnetic nanoparticles in 3d micrometastasic tumor tissue analogs for triple negative breast cancer. Biomaterials.

[64] Youngren-Ortiz S.R., Hill D.B., Hoffmann P.R., Morris K.R., Barrett E.G., Forest M.G., Chougule M.B. (2017). Development of optimized, inhalable, gemcitabine-loaded gelatin nanocarriers for lung cancer. J. Aerosol Med. Pulm. Drug Deliv..

[65] Sadhukha T., Wiedmann T.S., Panyam J. (2013). Inhalable magnetic nanoparticles for targeted hyperthermia in lung cancer therapy. Biomaterials.

[66] Karakoti A.S., Shukla R., Shanker R., Singh S. (2015). Surface functionalization of quantum dots for biological applications. Adv. Colloid Interface Sci..

[67] Zhang M., Wang W., Zhou N., Yuan P., Su Y., Shao M., Chi C., Pan F. (2017). Near-infrared light triggered photo-therapy, in combination with chemotherapy using magnetofluorescent carbon quantum dots for effective cancer treating. Carbon.

[68] Naasani I. (2016). Fighting cancer with quantum dots. IEEE Spectr..

[69] Torres-Lugo M., Rinaldi C. (2013). Thermal potentiation of chemotherapy by magnetic nanoparticles. Nanomedicine.

[70] Maeda H., Nakamura H., Fang J. (2013). The epr effect for macromolecular drug delivery to solid tumors: Improvement of tumor uptake, lowering of systemic toxicity, and distinct tumor imaging in vivo. Adv. Drug Deliv. Rev..

[71] Garcia Calavia P., Chambrier I., Cook M.J., Haines A.H., Field R.A., Russell D.A. (2018). Targeted photodynamic therapy of breast cancer cells using lactose-phthalocyanine functionalized gold nanoparticles. J. Colloid Interface Sci..

[72] Sawyers C. (2004). Targeted cancer therapy. Nature.

[73] (2016). What Is Targeted Cancer Therapy? American Cancer Society. https://www.cancer.org/treatment/treatments-and-side-effects/treatment-types/targeted-therapy/what-is.html.

[74] Xu X., Ho W., Zhang X., Bertrand N., Farokhzad O. (2015). Cancer nanomedicine: From targeted delivery to combination therapy. Trends Mol. Med..

[75] Liu J., Cui L., Losic D. (2013). Graphene and graphene oxide as new nanocarriers for drug delivery applications. Acta Biomater..

[76] Vishwakarma S.K., Sharmila P., Bardia A., Chandrakala L., Raju N., Sravani G., Sastry B.V.S., Habeeb M.A., Khan A.A., Dhayal M. (2017). Use of biocompatible sorafenib-gold nanoconjugates for reversal of drug resistance in human hepatoblatoma cells. Sci. Rep..

[77] National Cancer Institute at the National Institutes of Health (2017). Targeted Cancer Therapies. https://www.cancer.gov/about-cancer/treatment/types/targeted-therapies/targeted-therapies-fact-sheet.

[78] Amelio I., Melino G. (2015). The p53 family and the hypoxia-inducible factors (HIFs): Determinants of cancer progression. Trends Biochem. Sci..

[79] Ma Y., Huang J., Song S., Chen H., Zhang Z. (2016). Cancer-targeted nanotheranostics: Recent advances and perspectives. Small.

[80] Kytola V., Topaloglu U., Miller L.D., Bitting R.L., Goodman M.M., DAgostino R.B., Desnoyers R.J., Albright C., Yacoub G., Qasem S.A. (2017). Mutational landscapes of smoking-related cancers in caucasians and african americans: Precision oncology perspectives at wake forest baptist comprehensive cancer center. Theranostics.

[81] Lyer S., Wiekhorst F., Tietze R., Zaloga J., Janko C., Friedrich R., Cicha I., Engelhorn T., Struffert T., Schwarz M. Imaging and Quantification of Spions for Cancer Therapy with Magnetic Drug Targeting. Proceedings of the 2015 5th International Workshop on Magnetic Particle Imaging (IWMPI).

[82] Situ J.-Q., Wang X.-J., Zhu X.-L., Xu X.-L., Kang X.-Q., Hu J.-B., Lu C.-Y., Ying X.-Y., Yu R.-S., You J. (2016). Multifunctional SPIO/DOX-loaded A54 homing peptide functionalized dextran-g-PLGA micelles for tumor therapy and MR imaging. Sci. Rep..

[83] Yu M.K., Park J., Jon S. (2012). Targeting strategies for multifunctional nanoparticles in cancer imaging and therapy. Theranostics.

[84] Pathak S., Davidson M.C., Silva G.A. (2007). Characterization of the functional binding properties of antibody conjugated quantum dots. Nano Lett..

[85] Tran T.T., Tran P.H., Yoon T.J., Lee B.J. (2017). Fattigation-platform theranostic nanoparticles for cancer therapy. Mater. Sci. Eng. C Mater. Biol. Appl..

[86] Alric C., Taleb J., Duc G.L., Mandon C., Billotey C., Meur-Herland A.L., Brochard T., Vocanson F., Janier M., Perriat P. (2008). Gadolinium chelate coated gold nanoparticles as contrast agents for both x-ray computed tomography and magnetic resonance imaging. J. Am. Chem. Soc..

[87] Chen Y., Xianyu Y., Jiang X. (2017). Surface modification of gold nanoparticles with small molecules for biochemical analysis. Acc. Chem. Res..

[88] Su N., Dang Y., Liang G., Liu G. (2015). Iodine-125-labeled cRGD-gold nanoparticles as tumor-targeted radiosensitizer and imaging agent. Nanosci. Res. Lett..

[89] Popovtzer A., Mizrachi A., Motiei M., Bragilovski D., Lubimov L., Levi M., Hilly O., Ben-Aharon I., Popovtzer R. (2016). Actively targeted gold nanoparticles as novel radiosensitizer agents: An in vivo head and neck cancer model. Nanoscale.

[90] Shi Y., Yang S., Xing D. (2017). Quantifying the plasmonic nanoparticle size effect on photoacoustic conversion efficiency. J. Phys. Chem. C.

[91] Dou Y., Guo Y., Li X., Li X., Wang S., Wang L., Lv G., Zhang X., Wang H., Gong X. (2016). Size-tuning ionization to optimize gold nanoparticles for simultaneous enhanced CT imaging and radiotherapy. ACS Nano.

[92] Deng H., Zhong Y., Du M., Liu Q., Fan Z., Dai F., Zhang X. (2014). Theranostic self-assembly structure of gold nanoparticles for nir photothermal therapy and x-ray computed tomography imaging. Theranostics.

[93] Jeynes J.C.G., Geraki K., Jeynes C., Zhaohong M., Bettiol A.A., Latorre E., Harries L.W., Soeller C. (2017). Nanoscale properties of human telomeres measured with a dual purpose x-ray fluorescence and super resolution microscopy gold nanoparticle probe. ACS Nano.

[94] Liu T.-M., Conde J., Lipiński T., Bednarkiewicz A., Huang C.-C. (2017). Smart nir linear and nonlinear optical nanomaterials for cancer theranostics: Prospects in photomedicine. Prog. Mater. Sci..

[95] Gubin M.M., Artyomov M.N., Mardis E.R., Schreiber R.D. (2015). Tumor neoantigens: Building a framework for personalized cancer immunotherapy. J. Clin. Investig..

[96] Yu M., Zheng J. (2015). Clearance pathways and tumor targeting of imaging nanoparticles. ACS Nano.

